# Metastasis-Related Signature for Clinically Predicting Prognosis and Tumor Immune Microenvironment of Osteosarcoma Patients

**DOI:** 10.1007/s12033-023-00681-7

**Published:** 2023-02-20

**Authors:** Qing Zhang, Zhiping Deng, Yongkun Yang

**Affiliations:** Department of Orthopaedic Oncology Surgery, Beijing Jishuitan Hospital, Peking University, No 31, Xinjiekou Dongjie, Beijing, China

**Keywords:** Metastasis related genes, Prognostic biomarker, Function enrichment, Immune infiltration

## Abstract

Osteosarcoma is the most prevalent clinical malignant bone tumor in adolescents. The prognosis of metastatic osteosarcoma is still very poor. The aim of our study was to investigate the clinical diagnosis and prognostic significance of metastasis related genes (MRGs) in patients with osteosarcoma. Clinical information and RNA sequencing data with osteosarcoma patients were obtained and set as the training set from UCSC databases. GSE21257 were downloaded and chosen as the verification cohort. An eight gene metastasis related risk signature including MYC, TAC4, ABCA4, GADD45GIP1, TNFRSF21, HERC5, MAGEA11, and PDE1B was built to predict the overall survival of osteosarcoma patients. Based on risk assessments, patients were classified into high- and low-risk groups. The high-risk patients had higher risk score and shorter survival time. ROC curves revealed that this risk signature can accurately predict survival times of osteosarcoma patients at the 1-, 2-, 3-, 4- and 5- year. GSEA revealed that MYC targets, E2F targets, mTORC1 signaling, Wnt /β-catenin signaling and cell cycle were upregulated, and cell adhesion molecules, and primary immunodeficiency were decreased in high-risk group. MRGs were highly linked with the tumor immune microenvironment and ICB response. These results identified that MRGs as a novel prognostic and diagnostic biomarker in osteosarcoma.

**Supplementary Information:**

The online version contains supplementary material available at 10.1007/s12033-023-00681-7.

## Introduction

The most frequent malignant bone tumor is osteosarcoma in adolescents. Osteosarcoma remains the second leading cause of mortality in adolescents [24]. Osteosarcoma mainly occurs in the long bone metaphysis of the limb, and is commonly found in the distal humerus (43%), proximal tibia (23%), and proximal humerus (10%). The age of patients has an impact on the prevalence of osteosarcoma [40]. Clinically detectable distant metastases are found in 15%–20%. Metastasis can be detected in 10%–20% of patients with confirmed osteosarcoma [22]. The most common site of osteosarcoma metastasis is the lung, and patients with lung metastases from osteosarcoma have a recurrence risk of up to 80%. It is generally accepted that metastasis has a significant role in determining a cancer patient’s prognosis. Therefore, metastasis related genes are promising as new potential therapeutic targets for osteosarcoma.

Tumor metastasis is a very complex process modulated by metastasis related genes (MRGs) [7, 13]. Previous studies have demonstrated that MRGs exhibited strong prognostic potential in many tumors, including colorectal cancer [39], breast cancer [43], ovarian cancer [46], bladder cancer [42]. For example, 5-MRGs were identified to have strong prognostic and diagnosis biomarkers for the survival of melanoma based on gene expression datasets [35]. A 4-MRGs were identified to be a reliable and useful prognostic tool for the survival of breast cancer patients [43]. The high expression of metastasis related MAGEA11 is a worse prognosis in esophageal squamous cell carcinoma [12]. Increasing studies have proved that osteosarcoma metastasis related genes potentially participated in osteosarcoma metastasis progression, including IGFBP5, MMP11, FXYD2 and et al. [34, 36]. However, the diagnosis and prognosis of metastasis related gene in osteosarcoma is not yet complete elucidated.

Recently, numerous studies have shown that the tumor microenvironment (TME) plays an important role in the development and metastasis progression of cancer [50]. Various molecules and cells in the TME have complex and diverse effects on the occurrence, development and immunotherapy response of tumors [3]. The immune and inflammatory factor in TME play a key role in the efficacy of immunotherapy [25]. The rational design of tumor microenvironment activated nanocomposites provides an innovative strategy for constructing responsive tumor therapy [21, 47, 48].

In the present study, the aim of the study was to discover a novel metastasis associated prognostic biomarkers for osteosarcoma. A reliable signature was established rooted in MEGs, and its prognostic utility was systematically evaluated in OS patients. Additionally, the underlying connotations between the signature and the landscape of TME, namely, predictive enrichment of tumor infiltrating immune cells, and the expression level of immune checkpoints were explored, which offered novel insights for personalized immunotherapy. This was done by developing a risk score model based on MRGs to assess the prognostic value and immunotherapy efficiency of MRGs for osteosarcoma patients.

## Materials and Methods

### Collection of Osteosarcoma Datasets

Clinical data, count, and FPKM data were acquired from UCSC xena (https://xenabrowser.net/datapages/), the training cohort included 85 osteosarcoma samples. 47 osteosarcoma samples (GSE21257) were acquired from (GEO; https://www.ncbi.nlm.nih.gov/geo/) database and used as the verification cohort.

### Screening Metastasis Related Differentially Expressed Genes

The osteosarcoma patients were placed into two groups from TARGET databases, including metastasis and non-metastasis group. The DEGs were analyzed by used DEseq2 from the training set. *p*. adjust values <  = 0.05 and |log2FC|> = 1 [18].

### Construction of Metastasis Related Prognostic Signature

Genes that substantially associated with patient prognosis were identified by a univariate Cox proportional HR analysis in the training set. The “glmnet” R package (version 4.1.1) was used to perform LASSO regression to prevent model overfitting [9]. The risk score formula looked for genes with independent prognostic values. Next, the DEGs were found in between the high-risk and low-risk groups by R package Limma (version 3.42.2).

### Diagnostic Curve ROC Analysis

Kaplan–Meier curves was analyzed by using the “survival” R packages (version 3.2.10). We used the “pROC” (version 1.18.0) to create a time dependent ROC curve to assess the risk score’s efficacy in predicting the 1, 2, 3, 4, and 5-year survival of osteosarcoma patients.

### Functional Analyses and Mechanism Exploration

To determine the molecular basis of the prognostic gene, GSEA was performed in accordance with the Molecular Signatures Database (MSigDB, version 7.1.symbols.gmt). We decided to analyze the “KEGG gene sets (c2.cp.kegg.v7.1.symbols.gmt)” and “HALLMARK gene set (h.all.v7.1.symbols.gmt)”.

### Cibersort Analysis

The CIBERSORT [23] was used to analyze the abundance of 22 tumor immune infiltrating cell types in the tumor immune microenvironment (TME) of osteosarcoma patient samples. The heatmap was used to show the differentially abundance of 22 immune infiltrating cell. Wilcoxon test was used to statistically significant for the results of CIBERSORT analysis in high- and low-risk group.

### Immune Checkpoint Molecules Expression

The potential immunotherapeutic markers including 18 ICB-related genes were explored in high- and low-risk groups by Wilcoxon test. The heatmap of ICB-related genes expression was drawn by R package of “pheatmap”.

### Immunotherapeutic Response Prediction

ImmuCellAI platform (http://bioinfo.life.hust.edu.cn/web/ImmuCellAI/) was used to predict the response to immune checkpoint inhibitors in the sample of osteosarcoma patients [17].

### Statistical Analysis

All statistical analyses were conducted by R software. Wilcox test was used to the differentially analysis. *P* < 0.05 was considered statistical significance.

## Results

### Identification of DEGs in Metastasis and Non-metastasis Patients of Osteosarcoma

**Fig. 1:**
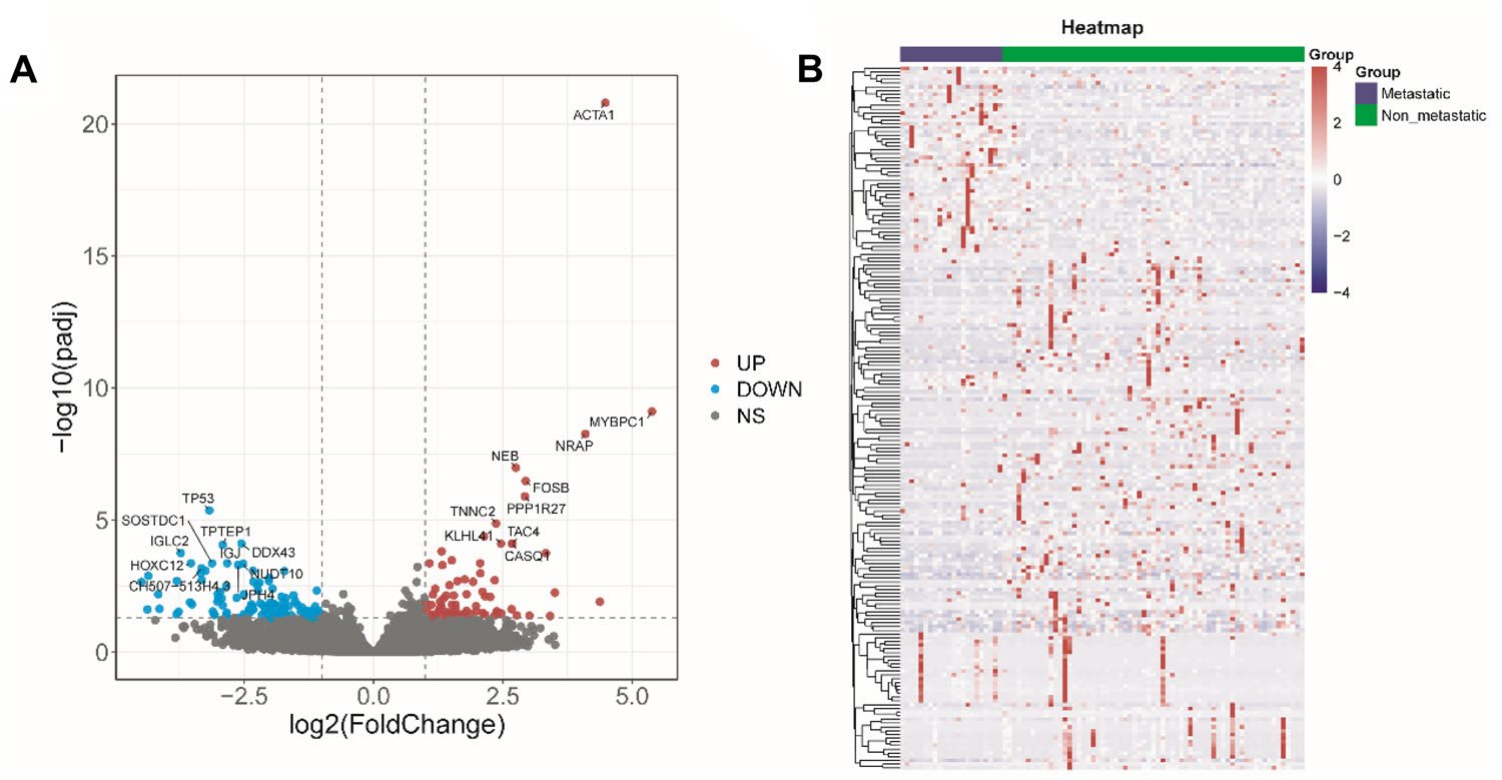
Differentially expressed genes in patients with metastatic and non-metastatic osteosarcoma. **A** Valcano plot of DEGs in metastatic group vs non-metastatic group. Downregulated genes are shown by blue dots, Up-regulated genes are represented by red dots. **B** Heatmap of significantly DEGs in metastatic group vs non-metastatic group

The clinical information and mRNA expression data were downloaded from the UCSC dataset. A total of 189 DEGs (70 upregulated and 119 downregulated) were identified in between metastatic group and non-metastatic group, including 127 coding protein genes and 62 non coding genes (Supplement Table 1). Volcano map showed that differentially expressed genes in metastatic group vs non-metastatic group (Fig. 1A). Blue dots represent downregulated genes, including TP53, TPTEP1, IGJ, DDX43, NUDT10, HOXC12, et al.; red dots represent up-regulated genes, including ACTA1, MYBPC1, NRAP, NEB, TNNC2, FOSB, et al. Heatmap revealed that the expression of DEGs in metastatic group and non-metastatic group (Fig. 1B). Red signifies higher expression, and blue signifies lower expression in metastatic group than non-metastatic group. DEGs in between metastasis group non-metastasis group of OS patients were identified.

### Construction of Metastasis Related Prognostic Model Based on the TARGET Dataset

**Fig. 2:**
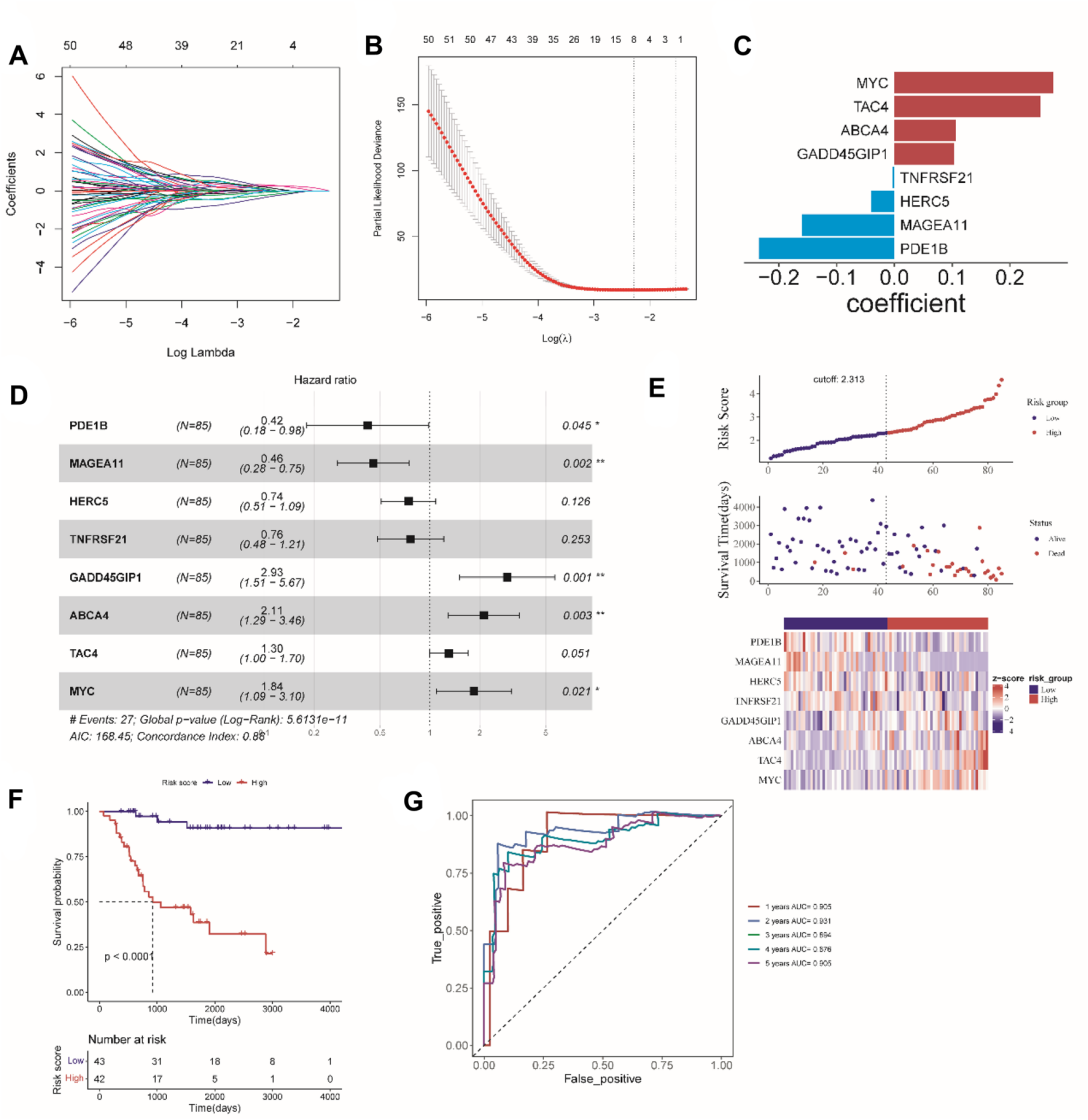
Construction of Metastasis related prognostic model in the training set. **A** LASSO coefficient profiles of 50 MRGs. **B** LASSO model with optimal lambda value. **C** LASSO coefficient configuration of eight candidate genes. **D** The forest plot of multivariate Cox regression. **E** Distribution of risk score and survival status, expression of eight candidate genes in osteosarcoma patients. **F** The Kaplan–Meier survival analysis of OS in TCGA cohort. **G** ROC analysis for risk score at 1, 2, 3, 4, 5 years

We used LASSO regression analysis to build a metastasis related prognostic model based on these 127 code-protein DEGs in the training set (Fig. 2A and B). A total of eight genes were acquired in the model, including MYC, TAC4, ABCA4, GADD45GIP1, TNFRSF21, HERC5, MAGEA11, and PDE1B (Fig. 2C). By using multivariate Cox proportional HR analysis, eight MRGs were found and used to create prognostic signature for patients OS (Fig. 2D). As shown in Fig. 2E, in accordance with the constructed prognostic model, each patient’s risk values were computed and patients were divided into high-risk or low-risk groups depending on the score; the patients of the low- risk group was longer survival time than the high-risk group. Next, the heat map showed that the expression of eight MRGs, PDE1B, MAGEA11, HERC5, and TNFRSF21 were high-expressed in the low-risk group, and GADD45GIP1, ABCA4, TAC4, and MYC were high-expressed in the high-risk group.

To explore the predictive value of the signature, the Kaplan–Meier survival curves were analyzed. As in Fig. 2F, the survival rate of high-risk group was lower than the low-risk group. ROC analysis confirmed that the area under the ROC curve more than 0.5 regardless of the predicted survival time at 1, 2, 3, 4, 5-year survival in the training set (Fig. 2G).

### Validation of the MRGs Related Prognostic Model in the External Databases

**Fig. 3:**
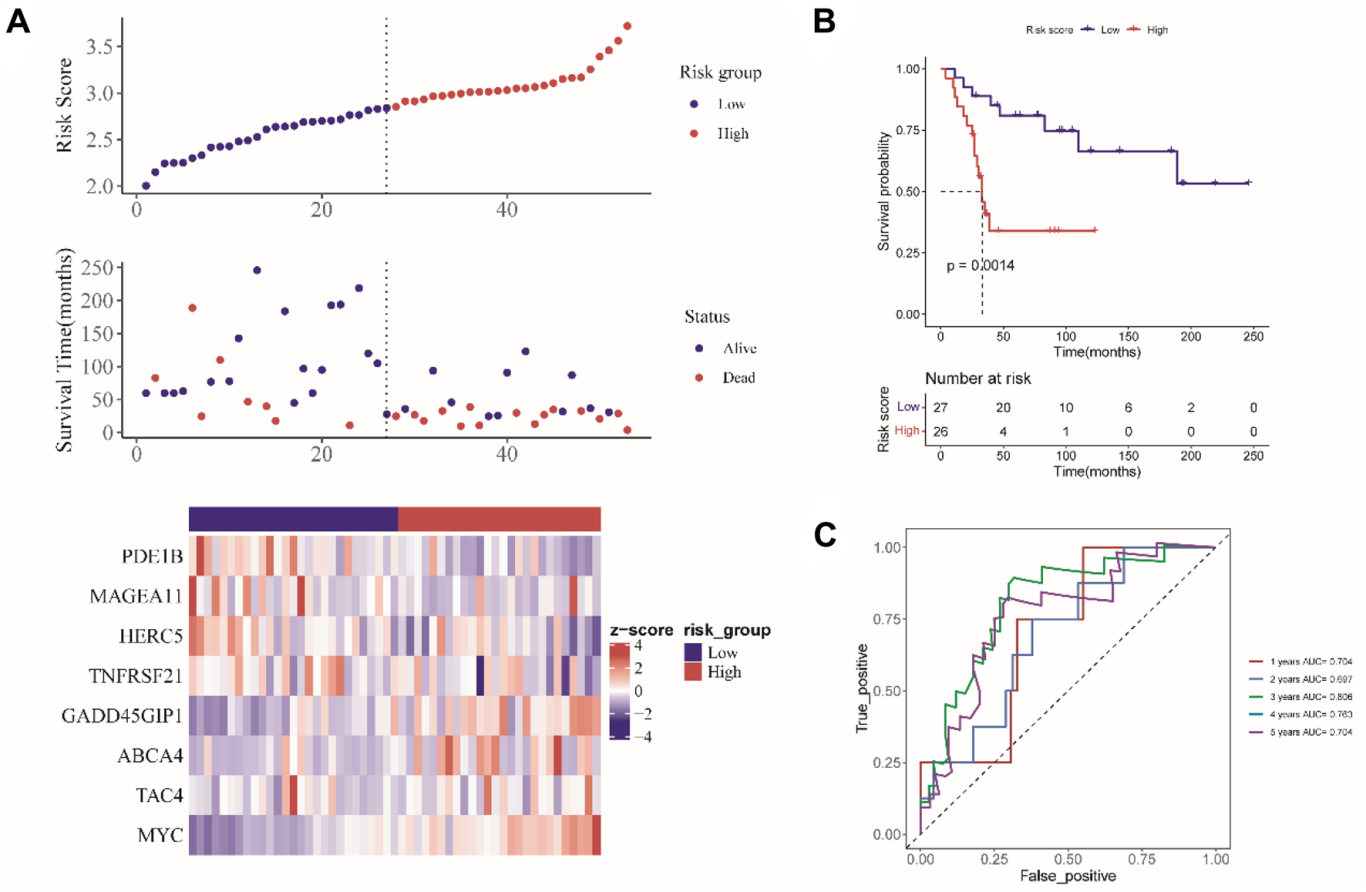
The constructed risk model is validated in the verification set. **A** survival status, and risk score, and expression of MRGs in osteosarcoma patients. **B** The Kaplan–Meier curve of OS. **C** ROC analysis for risk score at 1, 2, 3, 4, 5 years

The GEO dataset was used to validate the constructed prognostic model. As shown in Fig. 3A, patients in the GEO cohort were divided into 2 subgroups based on their risk scores calculated using the constructed prognostic model. The survival time of the low- risk group was longer than the high-risk group. Next, the heat map showed that the expression of eight MRGs, PDE1B, MAGEA11, HERC5, and TNFRSF21 were high-expressed in the low-risk group, and GADD45GIP1, ABCA4, TAC4, and MYC were high-expressed in the high-risk group. Survival analysis found that patients of the high-risk group have poor prognosis (Fig. 3B). Moreover, ROC analysis found that the AUC of 1, 2, 3, 4, 5-year survival was 0.704, 0.697, 0.806, 0.763, and 0.704, respectively (Fig. 3C). Above results collectively illustrated that the constructed prognostic model’s prediction accuracy.

### Functional Enrichment Analysis

**Fig. 4:**
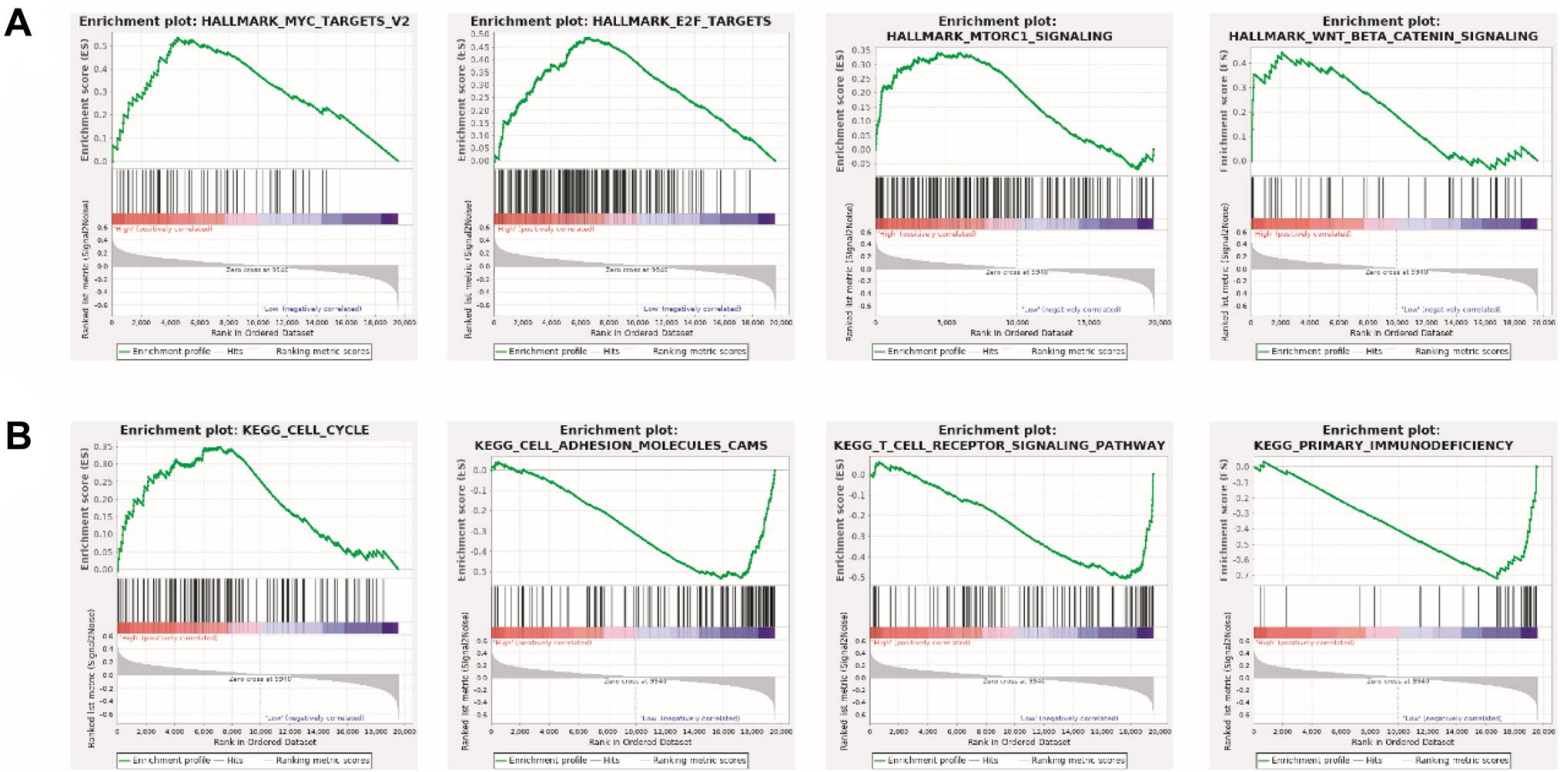
The potential molecular mechanism of the Metastasis related genes. **A** HALLMARK of Metastasis related genes score was analyzed by GSEA. **B** KEGG of Metastasis related genes score was analyzed and enriched by GSEA

We used GSEA to determine the enrichment pathways. HALLMARK pathways were mostly enriched in the MYC TARGETS, E2F TARGETS, MTORC1 signaling, and WNT β-catenin signaling (Fig. 4A). KEGG pathways of the high-risk group enriched in the Cell cycle; KEGG pathways of low-risk group enriched in the primary immunodeficiency, cell adhesion molecules, T cell receptor signaling pathway (Fig. 4B).

### Comparison of the Immune Microenvironment and the Immune Checkpoint Molecules Expression in the Between High-Risk and Low-Risk Groups

**Fig. 5:**
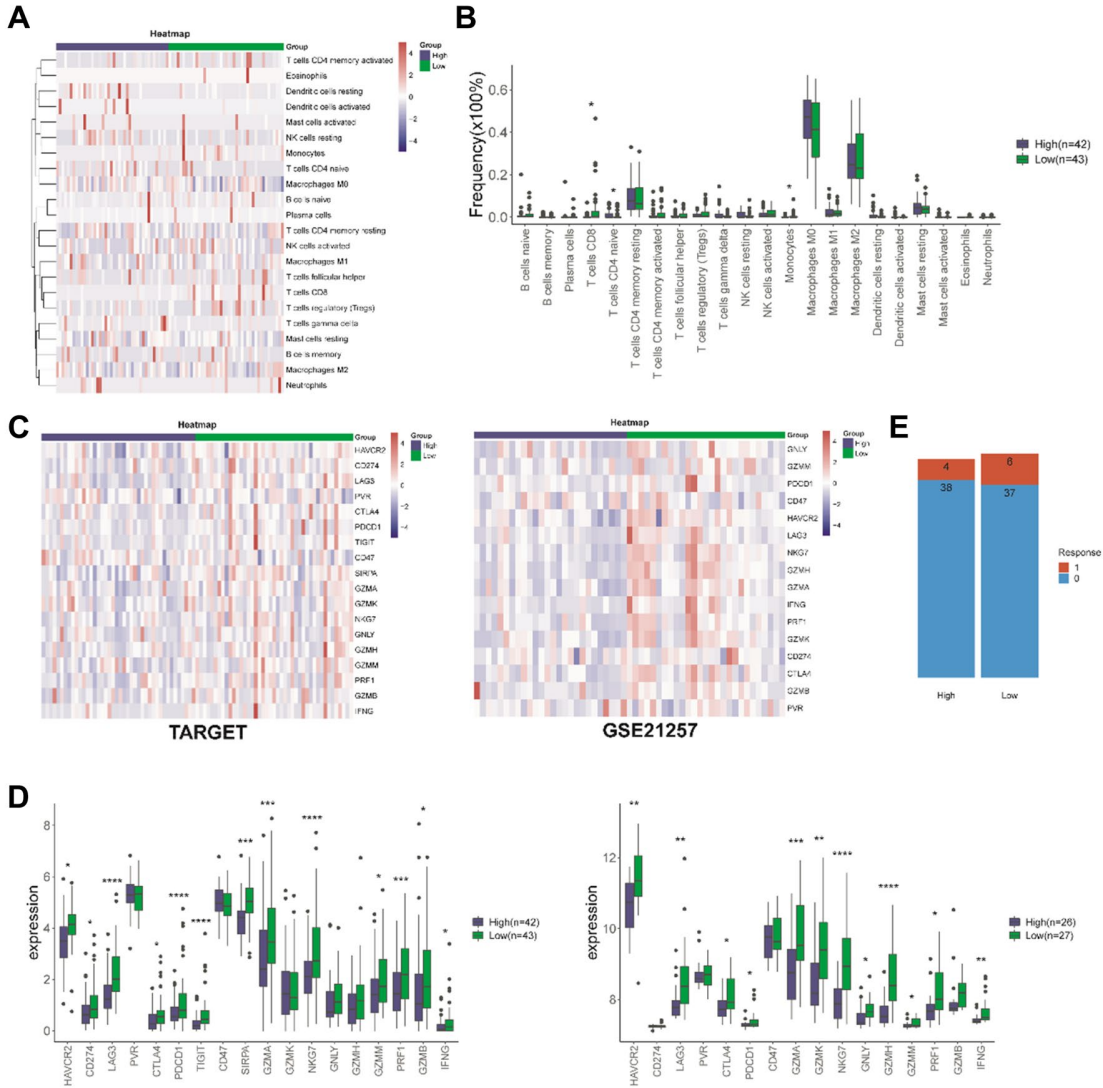
The landscape of TME. **A** The heatmap of tumor infiltrating immune cells expression. **B** Differentially analysis of 22 tumor infiltrating immune cells by Wilcoxon test analysis. **C** and **D** The heatmap of the expression of immune checkpoint molecules in low- and high-risk groups of TARGET and GSE21257. **E** The response to immune checkpoint inhibitors was predicted in osteosarcoma patient. (*P < 0.05; **P < 0.01; ***P < 0.001)

Metastasis is linked to immunity, we looked at the differences of immune infiltration in the between high-risk and low-risk groups. We employed CIBERSORT to analyze the difference of 22 immune cells in between high-risk and low-risk groups for each osteosarcoma patients. The heatmap of the tumor infiltrating immune cells (TIICs) expression was showed in high/low-risk groups (Fig. 5A). The composition of TIICs remained basically the same, mainly composed of T cells CD4 memory, Dendritic cells (DC), NK cells, Macrophages, and Mast cells. Wilcoxon test analysis showed T cells CD4 naive was higher infiltration, and B cells naïve, T cells CD8 and Monocytes were lower infiltration in the high-risk group than low-risk group (Fig. 5B).

Immune blocking checkpoint (ICB) related gene expression levels were correlated with therapeutic response of immune checkpoint inhibitors and targeted ICB checkpoints has emerged as promising strategy in cancers treatment [11, 26]. To better explore the potential of MRGs for predicting the response of osteosarcoma patients to immunotherapy, we analyzed the expression of immune checkpoint molecules in low-and high-groups of TRAGET and GSE21257. As shown in Fig. 5C and D, the expression of immunomodulators (HAVCR2, LAG3, CTLA4, PDCD1, GZMA, NKG7, GZMM, IFNG) were significantly increased in low-risk group than high-risk group in TRAGET dataset and GSE21257 dataset. Response to immune checkpoint inhibitors was predicted in each osteosarcoma patient sample, the patients of responded to immune checkpoint inhibitors in low-risk group were more than high-risk group (Fig. 5E).

## Discussion

Osteosarcoma is the most common malignant tumors with a strong ability for invasion and metastasis in bone tissue, which tends to occur in children and adolescent [30]. The osteosarcoma patients are associated with lung metastasis. The overall survival of OS patients has been still far away from satisfactory. High throughput sequencing technologies and bioinformatics analysis have recently used the investigation of genetic alterations in osteosarcoma and given a useful method to find potentially helpful markers [19, 27, 45]. In this present study, a total 197 DEGs were identified in between Metastasis group and Non-metastasis of osteosarcoma, including downregulated genes (TP53, TPTEP1, IGJ, DDX43, NUDT10, HOXC12, et al.) and up-regulated genes (ACTA1, MYBPC1, NRAP, NEB, TNNC2, FOSB, et al.).

Eight MRGs were developed prognostic model, including PDE1B, MAGEA11, HERC5, TNFRSF21, GADD45GIP1, ABCA4, TAC4, and MYC, all of them were risk factors in osteosarcoma. These MRGs have been reported as prognostic biomarkers in cancers. Metastasis related melanoma associated antigen-A11(MAGEA11) positive expression is an independent unfavorable prognostic factor in ESCC patients [29]. PDE1B was identified as potential prognostic biomarkers in osteosarcoma [32]. Weighted gene correlation network analysis identified HERC5 as prognostic candidate for breast cancer [33]. Lower expression of TNFRSF21 had a prominent advantage in survival and was correlated with a low level of immune infiltration in pancreatic adenocarcinoma [44]. Variation of ABCA4 was associated with therapy response in breast cancer [14]. Previous study has shown that TAC4 mRNA expression in gliomas, indicating a possible involvement of HK-1 in glioma biology [4]. MYC was overexpressed in osteosarcoma, and higher MYC expression related with metastasis and poor prognosis [8]. The high expression of CR6-interacting factor (CRIF1) is associated with unfavorable prognosis of hepatocellular carcinoma patients [6]. Almost all MRGs were highly related with cancer metastasis. As a result, 8 MRGs may be used as a metastasis related prognostic biomarker in a variety of therapeutic applications.

Epithelial-mesenchymal transition (EMT) is major factor contributing to the metastasis of cancer cells [2]. Consequently, the metastasis related signatures are appropriate therapeutic targets in the treatment of metastasis. Previous study has found that CRIF1 promoted hepatocellular carcinoma metastasis by inducing cell EMT [6]. Overexpression of c-Myc oncogene has been implicated in EMT in pancreatic cancer, [1, 51] lung cancer [52], and hepatocellular carcinoma [41].

We used GSEA analysis to investigate the possible molecular pathways linked with the high-risk group to better explored the underlying biological process. HALLMARK pathways were mainly enriched in the MYC TARGETS, E2F TARGETS, MTORC1 signaling, and WNT/β-catenin signaling. Previous studies have reported that Wnt/β-catenin signaling pathway was correlated with the lung metastasis of osteosarcomas [53]. It was reported that MYC was related to metastasis of patients with osteosarcoma [31]. LncRNA UCA1 promoted osteosarcoma metastasis by activating mTOR signaling pathways [20]. Therefore, the upregulated MYC TARGETS, E2F TARGETS, MTORC1 signaling, and WNT β-catenin signaling in high-risk group promoted metastasis of patients with osteosarcoma. KEGG pathways were involved in the cell cycle in the high-risk group. For example, LncRNA LINC01296 promoted cell proliferation and metastasis of osteosarcoma through regulating cell cycle protein cyclin D1 [49]. KEGG pathways of low-risk group involved in the cell adhesion molecules (CAMs). Mounting studies have revealed that epithelial mesenchymal transition is a most important process of tumor metastasis, the cell adhesion was weakened or disappeared to promote the invasion and migration of tumor cell [28, 37, 38].

Tumor microenvironment (TME) play a critical effect on tumors incidence and development. It has been reported that tumor associated macrophages promoted angiogenic stromal remodeling and linked to the progression and prognosis of osteoblastoma [5, 37, 38]. The results of this study showed that macrophages are the most important infiltrating immune cells in osteosarcoma, including undifferentiated M0 macrophages and M2 macrophages, and the role of M2 macrophages in osteosarcoma microenvironment needs to be further studied. Previous studies have reported that the higher level of Macrophages M0 and lower CD8T cells are associated with worst overall survival [16]. We demonstrated that the infiltration of T cells CD8 and monocytes were upregulated in high-risk patients. Therefore, the higher T cells CD8 and monocytes are associated with poor prognosis in osteosarcoma patients.

In the past decade, the ICB immunotherapies has achieved positive response in osteosarcoma patients [10]. However, only a small proportion of osteosarcoma patients can respond to immunotherapies, and the major reason might be the limitations in their tumor immunity status [15]. To verify whether MGR was capable of predicting the efficiency of anti-cancer immunotherapies in osteosarcoma patients, we analyzed the expression levels of immunomodulators (HAVCR2, LAG3, CTLA4, PDCD1, GZMA, NKG7, GZMM, IFNG) were significantly increased in low-risk group than high-risk group of TRAGET dataset and GSE21257 dataset. These results suggested that osteosarcoma patients in the low-risk group might have a better response to anti-CTLA4 and anti-PDCD1 antibodies. Besides, it is estimated that the low-risk group might respond better to immunotherapies than those in the high-risk group.

In this study, we explored the effect of MRGs on the genesis, development of OS through comprehensive bioinformatic analysis. We identified high-risk and low-risk groups of OS patients based on their MRGs expression matrix, and high-risk and low-risk groups showed different immune status and prognosis. We developed a prognosis risk model for better prediction of OS patient survival. The findings of this study could provide a new perspective and direction for future research on molecular targeted therapy of OS. Despite MRGs were identified to predict the survival of osteosarcoma, our study has several limitations. First, this study is based on bioinformatics methods for analysis and interpretation, the exact conclusion still needs to be verified by further experiments. Second, the number of samples used in this investigation is restricted. Third, the lack of experiment validation limited the evidence level of this study. Next, more research will be required to investigate the molecular mechanism. The role of MRGs in OS cell, which will be addressed in future studies.

## Conclusion

In conclusion, we analyzed differentially expressed mRNAs in between metastasis and non-metastasis osteosarcoma. We discovered a novel 8 MRGs in the diagnosis and prognosis of osteosarcoma patients.

### Supplementary Information

Below is the link to the electronic supplementary material.

Supplementary file1 (XLSX 23 KB)

## Data Availability

The datasets analyzed during the current study are available from the corresponding author on reasonable request.
